# FOXD1 expression in head and neck squamous carcinoma: a study based on TCGA, GEO and meta-analysis

**DOI:** 10.1042/BSR20210158

**Published:** 2021-07-28

**Authors:** Junjie Huang, Bin Liang, Tianjiao Wang

**Affiliations:** Department of Bioinformatics, Key Laboratory of Cell Biology, Ministry of Public Health, and Key Laboratory of Medical Cell Biology, Ministry of Education, School of Life Sciences, China Medical University, Shenyang 110122, China

**Keywords:** bioinformatics, Head and neck cancer, Immune, Prognosis, Tumor environment

## Abstract

Forkhead box D1 (*FOXD1*) is a new member of FOX transcription factor family. *FOXD1* has demonstrated multilevel roles during normal development, and several diseases’ pathogenesis. However, little is known about the role of FOXD1 in the progression of head and neck squamous cancer (HNSC). In the present study, we analyzed *FOXD1* expression pattern using The Cancer Genome Atlas (TCGA) dataset, Gene Expression Omnibus (GEO) datasets, HNSC cell lines, and HNSC tissues. Then, we analyzed the correlation between *FOXD1* expression and clinical characteristics, and evaluated the prognostic value of *FOXD1* in HNSC. Moreover, we assessed the relationship between *FOXD1* expression and tumor microenvironment (TME) and immune cell infiltration using Estimation of STromal and Immune cells in MAlignant Tumor tissues using Expression data (ESTIMATE) and Cell-type Identification By Estimating Relative Subsets Of known RNA Transcripts (CIBERSORT) algorithms. Finally, we predicted the *FOXD1*-related biological processes (BPs) and signal pathways. *FOXD1* was up-regulated in HNSC tissues in TCGA datasets, validated by GEO datasets, HNSC cell lines and HNSC tissues. FOXD1 expression was significantly associated with tumor site and HPV infection. Univariate and multivariate Cox regression analyses showed that *FOXD1* expression was an independent prognostic factor. Moreover, we found that the proportions of naïve B cells, plasma cells, and resting dendritic cells (DCs) were negatively correlated with *FOXD1* expression, otherwise, the proportion of activated mast cells was positively correlated with *FOXD1* expression using CIBERSORT algorithm. Gene Set Enrichment Analyses (GSEAs) revealed that *FOXD1* was mainly involved in cancer-related signaling pathway and metabolism-related pathways. *FOXD1* was a potential oncogene, and might represent an indicator for predicting overall survival (OS) of HNSC patients. Moreover, many cancer-related pathways and metabolism-related processes may be regulated by FOXD1.

## Introduction

Head and neck cancer (HNC) is an aggressive malignant tumor arising in oral cavity, oropharynx, hypopharynx and larynx, representing the sixth most common cancer and fifth leading cause of cancer-related deaths worldwide [1]. It is estimated that more than 51000 new cases of HNC were diagnosed in 2018, resulting in an annual incidence of 10000 deaths in United States [2]. The vast majority of HNC are squamous carcinomas (head and neck squamous cancer, HNSC), accounting for more than 90% of HNC. Over the past 30 years, even if the combination of surgery, chemotherapy, radiotherapy and novel immune checkpoint inhibitors have been applied, the 5-year survival rate for HNSC, especially for advanced HNSC patients, has not yet been remarkably improved. Growing evidence has demonstrated that HNSC is a highly complex and heterogeneous disease, involving distinct histological types, different anatomical sites and varied genetic and molecular alterations, thus, hampering the clinicians to accurately diagnose, guide individualized treatment, and improve the prognosis of HNSC patients. Therefore, there is still an urgent need to explore the effective biomarkers and underlie the molecular mechanism in the development and progression of HNSC.

Forkhead box D1 (*FOXD1*), also known as *FKHL8, FREAC-4* and *FREAC4*, is a new member of FOX transcription factor family and located on 5q13.2. *FOXD1* has demonstrated multilevel roles during normal development, adult physiology, and several diseases’ pathogenesis. Previous studies reported that *FOXD1* was a mediator and indicator in the process of cell programming, especially in kidney development [3–5]. Herrera et al. reported that *foxd1* plays a dual role in retinal ganglion cell axon migration through the optic chiasm [6]. Moreover, accumulating evidence has demonstrated that FOXD1 is implicated in the carcinogenesis, including lung cancer [7], colorectal cancer [8], ovarian cancer [9], breast cancer [10], nasopharyngeal carcinoma [11], gastric cancer [12], and prostate cancer [13]. Gao et al. also found that FOXD1 was up-regulated and directly correlated with the glioma grade, and regulated glioblastoma cell behaviors [14]. However, little is known about the role of FOXD1 in the progression of HNSC.

In the present study, we analyzed *FOXD1* expression pattern using The Cancer Genome Atlas (TCGA) and Gene Expression Omnibus (GEO) datasets. First, we investigated the *FOXD1* expression between HNSC tissues and normal tissues, analyzed the correlation between *FOXD1* expression and clinical characteristics, and evaluated the prognostic value of *FOXD1*. Moreover, we assessed the relationship between *FOXD1* expression and tumor microenvironment (TME) and immune cell infiltration using Estimation of STromal and Immune cells in MAlignant Tumor tissues using Expression data (ESTIMATE) and Cell-type Identification By Estimating Relative Subsets Of known RNA Transcripts (CIBERSORT) algorithms. Accordingly, the relationship between *FOXD1* expression and underlying biological functions and signal pathways in HNSC was analyzed using Gene Set Enrichment Analysis (GSEA) tool. Together, our results could provide a new insight into the potential mechanism contributing to HNSC development and progression, and highlight new targets for clinical treatment.

## Materials and methods

### Overview of *FOXD1* gene

*FOXD1* expressions were compared between cancer tissues and normal tissues across all TCGA tumors using TIMER online tool (http://timer.cistrome.org/). In addition, we analyzed the significant FOXD1-correlated genes using LinkedOmics online database (http://linkedomics.org). The LinkedOmics database contains multiomics data for 32 cancer types and a total of 11158 patients from TCGA project. Then, we evaluated the biological functions and pathways for *FOXD1* and *FOXD1*-correlated genes through STRING database (https://string-db.org/).

### Data collection

The RNA-sequencing data and related clinical information were downloaded from TCGA database (https://www.cancer.gov/tcga). The sample inclusion criteria: completed *FOXD1* sequencing data and detailed clinical information, including age, gender, TNM stage, follow-up information.

**Figure 1:**
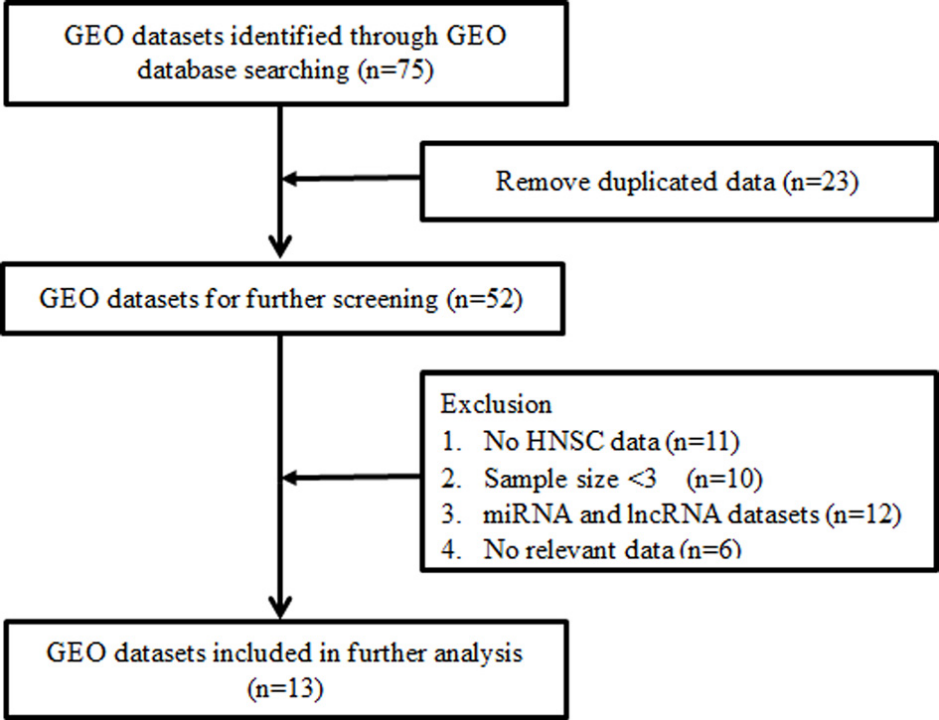
Flow chart of the GEO dataset selection process

The microarray HNSC datasets were searched from GEO database (http://www.ncbi.nlm.nih.gov/geo/) up to December 2020. The search terms included the following keywords: (head OR neck OR oral) AND (tumor OR cancer OR carcinoma OR neoplasm). Each GEO dataset should meet the following criteria: (1) completed *FOXD1* expression data; (2) study including HNSC and control group; (3) the number of each group was greater than three. The flow diagram of the GEO dataset selection is shown in Figure 1. All characteristics of GEO datasets are listed in Table 1.

**Table 1:** The characteristics of selected GEO datasets of HNSC

Datasets	Contributor (year)	Disease type	Experimental platform	Number of cases (cancer/control)
GSE6631	Kuriakose et al.(2007)	Head and neck squamous cell carcinoma	Affymetrix Human Genome U95 Array	22/22
GSE19089	Tuch et al*.* (2009)	Oral cancer	Illumina HumanHT-12 V3.0 beadchip	3/3
GSE23558	Ambatipudi et al. (2011)	Oral squamous cell carcinoma	Agilent-014850 Human Genome Microarray	27/5
GSE25099	Peng et al*.* (2011)	Oral squamous cell carcinoma	Affymetrix Human Exon 1.0 ST Array	57/22
GSE30784	Chen et al*.* (2011)	Oral squamous cell carcinoma	Affymetrix Human Genome U133 Array	167/45
GSE31056	Reis et al. (2011)	Oral squamous cell carcinoma	Affymetrix Human Genome U133 Array	23/24
GSE74530	Oghumu et al. (2017)	Oral squamous cell carcinoma	Affymetrix Human Genome U133 Array	6/6
GSE78060	Enokida et al. (2017)	Tongue squamous cell carcinoma	Affymetrix Human Genome U133 Array	26/4
GSE85195	Bhosale et al. (2017)	Oral squamous cell carcinoma	Agilent-014850 Human Genome Microarray	34/16
GSE13601	Singh et al. (2008)	Oral tongue cancer	Affymetrix Human Genome U95 Array	31/26
GSE31405	Rentoft et al. (2011)	Tongue carcinoma	Illumina HumanHT-12 WG-DASL V4.0 R2 beadchip	62/16
GSE34106	Rentoft et al. (2011)	Tongue carcinoma	Illumina HumanHT-12 WG-DASL V4.0 R2 beadchip	28/15
GSE146483	Kase (2020)	Oral squamous carcinoma	Agilent-039494 SurePrint G3 Human GE v2 8×60K Microarray 039381	8/3

### *FOXD1* expression patterns in HNSC patients

*FOXD1* expressions were compared between HNSC tissues and normal tissues in TCGA and GEO datasets. We also compared *FOXD1* expression between paired normal and tumor tissues. Moreover, the associations between *FOXD1* expression and clinical parameters were evaluated using TCGA dataset, including age, gender, distant metastasis, clinical stage, T stage, N stage and HPV status. A *P*<0.05 was considered significant.

### Association of *FOXD1* expression and clinical outcomes

Kaplan–Meier curves were used to evaluate the correlation between *FOXD1* expression and overall survival (OS), tested by Log-Rank test. Univariate and multivariate Cox regression analyses were performed to evaluate the independent prognostic value of *FOXD1*, as well as age, gender, distant metastasis, clinical stage, N stage, and T stage in HNSC. All independent prognostic parameters identified by multivariate Cox regression analyses were used to construct a nomogram to investigate the probability of 3- and 5-year OS of HNSC. The concordance index (C-index) was calculated to quantify the discrimination performance of this nomogram. The calibration curve was plotted to assess whether the predict probability was in agreement with actual rate in the nomogram.

### Relationship of *FOXD1* expression and TME

TME provides a more complex matrix around tumor cells, which contributes to tumor development, progression, metastasis, and drug resistance [15]. We adopted ESTIMATE method to calculate the stromal score and immune score, inferring the tumor purity for TCGA dataset. The stromal score and immune score were compared between high- and low- *FOXD1* expression group. Moreover, the CIBERSORT algorithm was performed to analyze the transcript data from TCGA dataset and calculate the proportions of immune infiltrating cell subtypes. Moreover, the correlation between *FOXD1* expression and the proportion of immune cell subtype was evaluated by Pearson correlation coefficient.

### GSEA

GSEA was employed to identify the underlying biological functions and pathways of high- and low-FOXD1 expression groups. The reference gene sets ‘c2.cp.kegg.v7.2.symbols.gmt gene sets’ and ‘C5.go.bp.v7.2.symbols.gmt gene set’, were obtained from the Molecular Signatures Database (MSigDB). The nominal *P*-value <0.05 and normalized enrichment score (NES) < 25% were considered significant.

### Cell lines

The human normal oral epithelial cells HOEC were obtained from Keygen Company Co., Ltd. HNSC cell lines CAL-27, SCC-9, and TCA-8113 were purchased from ATCC. HOEC and HNSC cell lines were cultured in 1640 and DMEMs containing 10% fetal bovine serum (Thermo Fisher Scientific, Waltham, MA) at 37°C and 5% CO_2_ in an incubator, separately.

### Real‐time polymerase chain reaction

Total RNA was extracted by TRIzol reagent (Invitrogen) and cDNA was synthesized using PrimeScript™ RT reagent Kit with gDNA Eraser (TaKaRa, Japan). The real-time polymerase chain reaction (RT-qPCR) was performed to determine RNA level using SYBR Green PCR Kit (TaKaRa, Japan). The primer sequences for the detection of *FOXD1* were 5′-TGAGCACTGAGATGTCCGATG-3′ (forward primer) and 5′-CACCACGTCGATGTCTGTTTC-3′ (reverse primer). Glyceraldehyde‐3‐phosphate dehydrogenase (GAPDH) was amplified to normalize *FOXD1* levels. The expression level was calculated by 2^−ΔΔ*C*_t_^ method. Each sample was measured in triplicates.

### Western blotting

Cells were lysed with RIPA Buffer (Beyotime Biotechnology Institute). Denatured proteins were separated on 10% SDS/PAGE and transferred on to PVDF membranes. The blocked membranes were incubated with primary FOXD1 antibody (Absin Bioscience, Inc) at 4°C overnight, and then incubated with horseradish peroxidase-conjugated goat anti-rabbit secondary antibody (Cell Signaling Technology, Inc) at room temperature for 2 h. Protein band was visualized by enhanced chemiluminescence reagent (Thermo Fisher Scientific, Inc.).

### Immunohistochemical staining evaluation

Primary HNSC tissues were collected from HNSC patients, who underwent surgical resection in the School and Hospital of Stomatology, China Medical University. All patients received no preoperative chemotherapy, immune therapy, and radiation therapy. The study was approved by the Ethics Committee of China Medical University. All study involving human participants were in accordance with the Declaration of Helsinki. All the enrolled participants provided written informed consent.

Paraffin-embedded tissue blocks were sectioned into 4-µm-thick sections for immunohistochemistry. All samples were deparaffinized and rehydrated. The citrate buffer (pH 6.0) and 3% hydrogen peroxide were used for antigen retrieval and endogenous peroxidase activity blocking, respectively. Then, all slides were incubated with goat anti-FOXD1 polyclonal antibody (1:100, Abcam Company, #ab129324) overnight at 4°C, followed by incubating with rabbit anti-goat horseradish peroxidase (HRP)-conjugated secondary antibody at room temperature for 30 min. Finally, all slides were visualized using DAB Horseradish Peroxidase Color Development Kit (Maixin Co., Fuzhou, China).

### Statistical analysis

All data were analyzed using R software (version 3.6.3) and SPSS 22.0 (SPSS Inc., Chicago, IL, U.S.A.). All data were expressed as mean ± standard deviation (SD). Statistical differences were evaluated using Student’s *t* tests or one-way ANOVA. Survival analyses were evaluated using Kaplan–Meier curve and Log-rank test. The univariate and multivariate Cox regression analyses were performed to identify the potential prognostic values of FOXD1 and clinical parameters. Pearson correlation analysis was used to assess the relationship between FOXD1 expression and the proportion of immune cell subtype. *P*-value <0.05 was considered significant.

## Results

### Overview of *FOXD1* expression pattern and features

**Figure 2:**
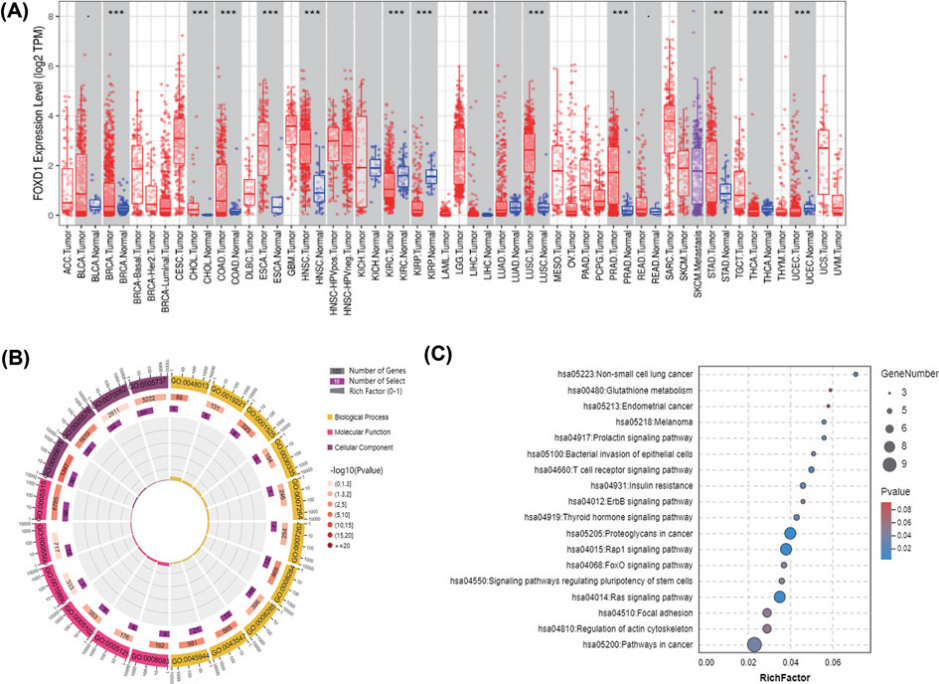
Pan-cancer analyses of *FOXD1* expression and functions of *FOXD1*-correlated genes in HNSC (**A**) Pan-cancer analyses of *FOXD1* expression using TIMER online tool. (**B**) BPs, CCs, and MFs of *FOXD1*-correlated genes based on TCGA dataset through STRING database. BPs: GO:0045944, positive regulation of transcription from RNA polymerase II promoter; GO:0043547, positive regulation of GTPase activity; GO:0008284, positive regulation of cell proliferation; GO:0008285, negative regulation of cell proliferation; GO:0001525, angiogenesis; GO:0007264, small GTPase-mediated signal transduction; GO:0007267, cell–cell signaling; GO:0030335, positive regulation of cell migration; GO:0000165, MAPK cascade; GO:0048013, ephrin receptor signaling pathway; GO:0019221, cytokine-mediated signaling pathway. CCs: GO:0005737, cytoplasm; GO:0070062, extracellular exosome; GO:0005576, extracellular region; GO:0005615, extracellular space. MFs: GO:0005515, protein binding; GO:0005509, calcium ion binding; GO:0008083, growth factor activity; GO:0005102, receptor binding; GO:0019899, enzyme binding; GO:0005125 cytokine activity. (**C**) KEGG pathways of *FOXD1*-correlated genes in HNSC through STRING database. * *P*-value<0.05, ** *P*-value<0.01, *** *P*-value<0.001.

We analyzed tumor samples from TCGA database to identify *FOXD1* expression characteristics (Figure 2A). *FOXD1* expression was up-regulated in breast invasive carcinoma (BRCA), cholangiocarcinoma (CHOL), colon adenocarcinoma (COAD), esophageal carcinoma (ESCA), head and neck squamous cell carcinoma (HNSC), liver hepatocellular carcinoma (LIHC), lung squamous cell carcinoma (LUSC), prostate adenocarcinoma (PRAD), and stomach adenocarcinoma (STAD). *FOXD1* expression was down-regulated in kidney renal clear cell carcinoma (KIRC), kidney renal papillary cell carcinoma (KIRP), thyroid carcinoma (THCA), and uterine corpus endometrial carcinoma (UCEC). Then, we used LinkedOmics tool to analyze the *FOXD1*-correlated genes (Supplementary Table S1) based on TCGA dataset and analyzed the GO functions and KEGG pathways with STRING database. The main GO functions and gene distributions, including biological process (BP), cell component (CC), and molecular function (MF), were shown in Figure 2B and Supplementary Table S2. The enrichment signal pathways were mainly distributed on proteoglycans in cancer, Rap1 signaling pathway, Ras signaling pathway, and T-cell receptor signaling pathway (Figure 2C).

### *FOXD1* was up-regulated in HNSC patients

**Figure 3:**
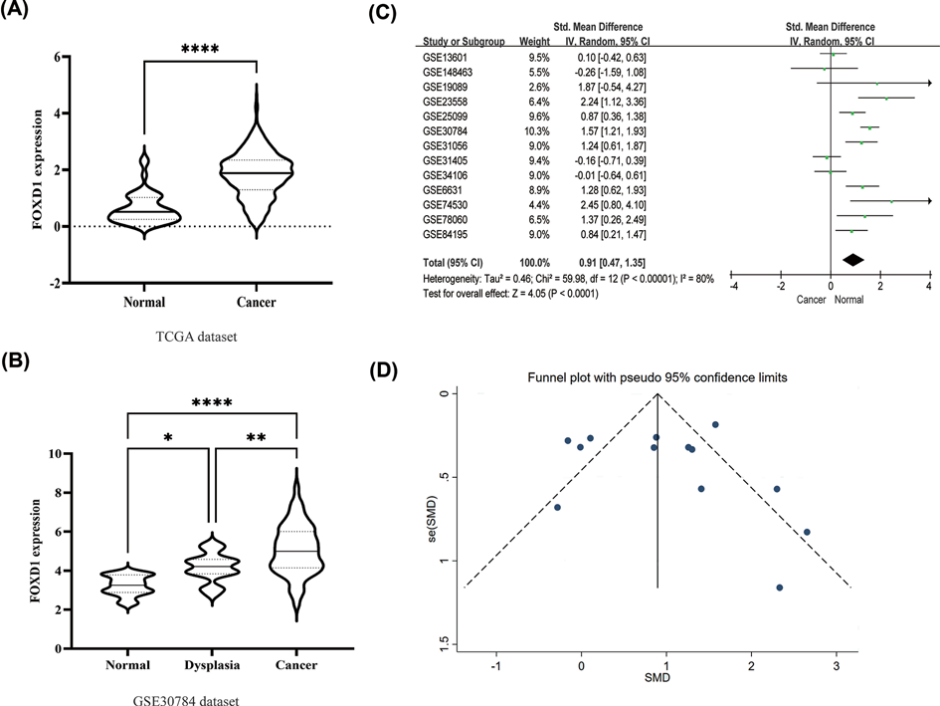
*FOXD1* expression in HNSC tissues (**A**) *FOXD1* expression in TCGA dataset. (**B**) *FOXD1* expression among HNSC, dysplasia, and normal tissues in GSE30784 dataset. (**C**) Comparisons of *FOXD1* expression between HNSC and normal tissues evaluated by forest-plot based on data from 13 GEO datasets. The random-effects model was adopted due to high heterogeneity (*I^2^* = 80.0%, *P* <0.001) for the included studies. The overall standard mean difference (SMD) was 0.91 (95% CI = 0.47–1.35), indicating that FOXD1 expression in HNSC tissues was significantly higher than normal tissues (**D**) The funnel plot of SMD for the included studies, indicating no publication bias. *P*<0.05 represented statistical significance. * *P*-value<0.05, ** *P*-value<0.01, **** *P*-value<0.0001.

According to inclusion criteria, a total of 449 HNSC samples and 44 normal samples from TCGA database were finally included in the study. The clinical characteristics were list in Supplementary Table S3. Compared with normal tissues, *FOXD1* expression was significantly up-regulated in HNSC tissues in TCGA dataset (*P*<0.001, Figure 3A). To validate the result reliability, we also compared *FOXD1* expression between paired HNSC tissues and adjacent normal tissues (*n*=43 pairs). *FOXD1* was also up-regulated in HNSC tissues than normal tissues (*P*<0.001, Supplementary Figure S1). Moreover, in GSE30784 dataset, *FOXD1* expression in HNSC tissues was significantly higher than benign dysplasia and normal tissues (*P*<0.001, Figure 3B).

Then, GEO datasets were as external validation. A total of 13 GEO datasets were included in the meta-analysis based on the inclusion criteria. These datasets were submitted from 2007 to 2020. FOXD1 expression in tumor tissues was significantly higher than normal tissues in GSE6631, GSE19089, GSE23558, GSE25099, GSE30784, GSE31056, GSE74530, GSE78060, and GSE85195 datasets (all *P*<0.05, Figure 3C). Meta-analyses indicated that the pooled standard mean difference (SMD) of *FOXD1* was 0.91 (*P*<0.001, 95% CI: 0.47–1.35) using random-effects model, indicating that *FOXD1* expression in HNSC was significantly higher than normal tissues. The funnel plot of SMD for the included studies appeared to be symmetric, and displayed no publication bias (Figure 3D).

### Association of FOXD1 expression and characteristics

**Figure 4:**
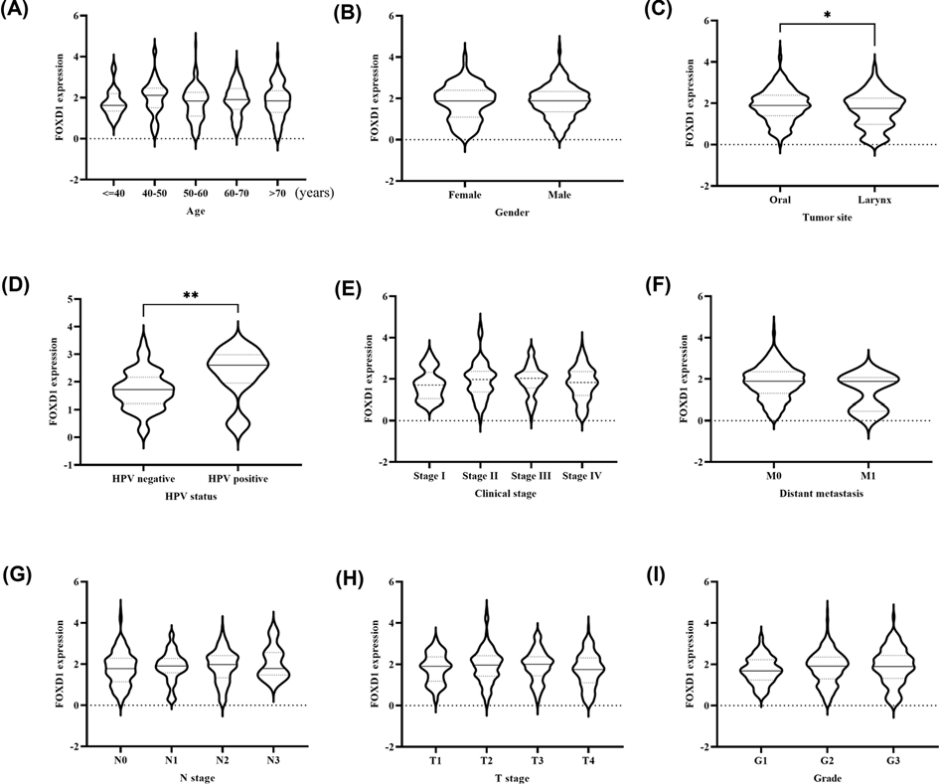
Correlation of *FOXD1* expression and clinical parameters (**A**) Age; (**B**) gender; (**C**) tumor sites; (**D**) HPV infection; (**E**) clinical stages; (**F**) distant metastasis; (**G**) N stage; (**H**) T stage; (**I**) Grade. *P*<0.05 represented statistical significance. * *P*-value<0.05, ** *P*-value<0.01.

In TCGA dataset, *FOXD1* expression was significantly associated with tumor site (*P*=0.010) and HPV infection (*P*=0.010). But, *FOXD1* expression was not associated with age (*P*=0.354), gender (*P*=0.483), distant metastasis (*P*=0.205), clinical stage (*P*=0.189), N stage (*P*=0.496), T stage (*P*=0.081), and grade (*P*=0.572) (Figure 4).

### Association of FOXD1 expression and clinical outcomes

**Figure 5:**
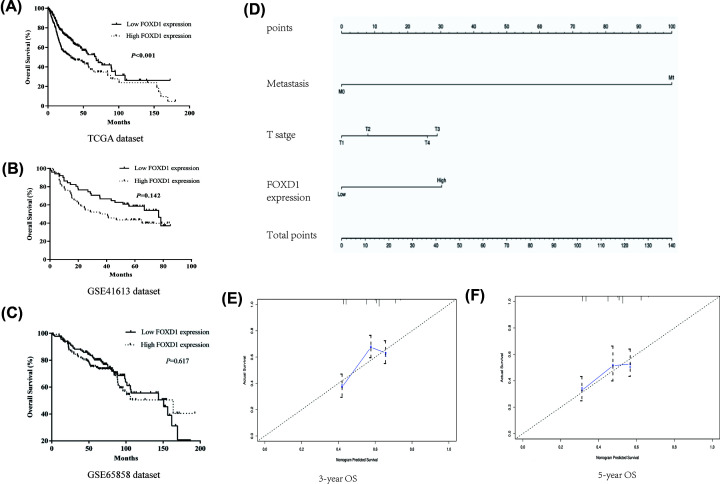
Prognostic analyses of *FOXD1* expression in HNSC patients (**A**) Kaplan–Meier curve of *FOXD1* expression in TCGA dataset. (**B**) Kaplan–Meier curve of *FOXD1* expression in GSE41613 dataset. (**C**) Kaplan–Meier curve of *FOXD1* expression in GSE65858 dataset. (**D**) Nomogram of OS predicting in HNSC patients. (**E,F**) The 3- and 5-year calibration curves for consistency validation of the nomogram.

In TCGA dataset, Kaplan–Meier curves showed that high *FOXD1* expression was significantly correlated with poor OS (OS: *P*<0.001, Figure 5A). GSE41613 and GSE65858 datasets were used as external validation. Although the difference was not significant in GSE41613 and GSE65858 datasets, high *FOXD1* expression had a tendency of low OS (Figure 5B,C). Univariate and multivariate Cox regression analyses showed that *FOXD1* expression was an independent prognostic factor for OS (*P*=0.001, Table 2). According to the result of multivariate Cox regression, the nomograms were generated to predict the 3- and 5-year OS probabilities, respectively. We introduced three independent factors into the nomogram for OS, including distant metastasis, T stage and *FOXD1* expression, and each factor was assigned a score in proportion to its contribution to the risk of survival, thus obtaining the corresponding predicted survival rate (Figure 5D). The C-index of OS prediction was 0.62. Calibration of the nomograms displayed good agreement between the predicted 3‐ and 5‐year OS rates and actual observations (Figure 5E,F).

**Table 2:** Univariate and multivariate Cox regression analyses of *FOXD1* in TCGA HNSC dataset

Parameters	Univariate analysis			Multivariate analysis		
	HR	95% CI	*P*-value	HR	95% CI	*P*-value
Age	1.259	0.952–1.665	0.106			
Gender	0.787	0.585–1.060	0.115			
Metastasis	4.516	1.663–12.265	0.003	5.947	2.154–16.423	0.001
Clinical stage	1.115	0.952–1.306	0.176			
N stage	1.178	1.013–1.370	0.033			
T stage	1.097	0.948–1.269	0.216	1.165	1.002–1.353	0.046
Grade	1.088	0.879–1.346	0.437			
Tumor site	0.899	0.651–1.242	0.520			
FOXD1 expression	1.608	1.216–2.1285	0.001	1.711	1.287–2.275	<0.001

### Relationship between *FOXD1* expression and TME

**Figure 6:**
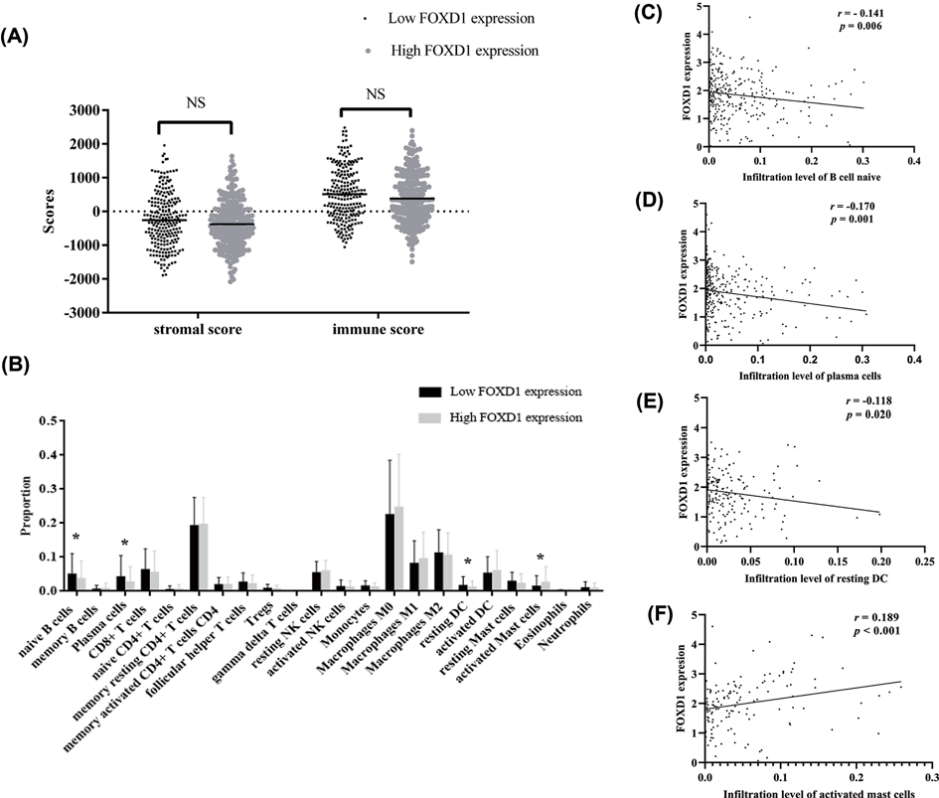
Correlation of *FOXD1* expression and TME and immune cell infiltration in HNSC (**A**) Correlation between *FOXD1* expression and stromal scores and immune scores between high- and low-*FOXD1* expression groups using ESTIMATE algorithm. (**B**) The proportions of immune cell subtypes between high- and low- *FOXD1* expression groups using CIBERSORT algorithm. (**C**–**F**) Correlations of naïve B cell, plasma cells, resting DC and activated mast cells, and FOXD1 expression in HNSC samples. *P*<0.05 represented statistical significance. * *P*-value<0.05.

To understand the immune characteristics of *FOXD1*, ESTIMATE was applied to calculate the stromal score and immune score in 449 HNSC patients. We found that stromal scores and immune scores displayed no significant difference between high- and low-*FOXD1* expression groups (*P*>0.05, Figure 6A). Then, the gene expression matrix of the HNSC dataset was analyzed using CIBERSORT algorithm to estimate the proportions of 22 immune infiltrating cells (Figure 6B). The proportions of naïve B cells (*P*=0.023), plasma cells (*P*=0.019), and resting dendritic cells (DCs) (*P*=0.018) in high *FOXD1* expression were significantly lower than low *FOXD1* expression group, while the proportion of activated mast cells in high *FOXD1* expression group was significantly higher than low *FOXD1* expression group (*P*=0.014). Moreover, we found that the proportions of naïve B cells (*r* = −0.141, *P*=0.006, Figure 6C), plasma cells (*r* = −0.170, *P*=0.001, Figure 6D), and resting DCs (*r* = −0.118, *P*=0.020, Figure 6E) were negatively correlated with *FOXD1* expression, otherwise, the proportion of activated mast cells was positively correlated with *FOXD1* expression (*r* = 0.189, *P*<0.001, Figure 6F).

### GSEAs

**Figure 7:**
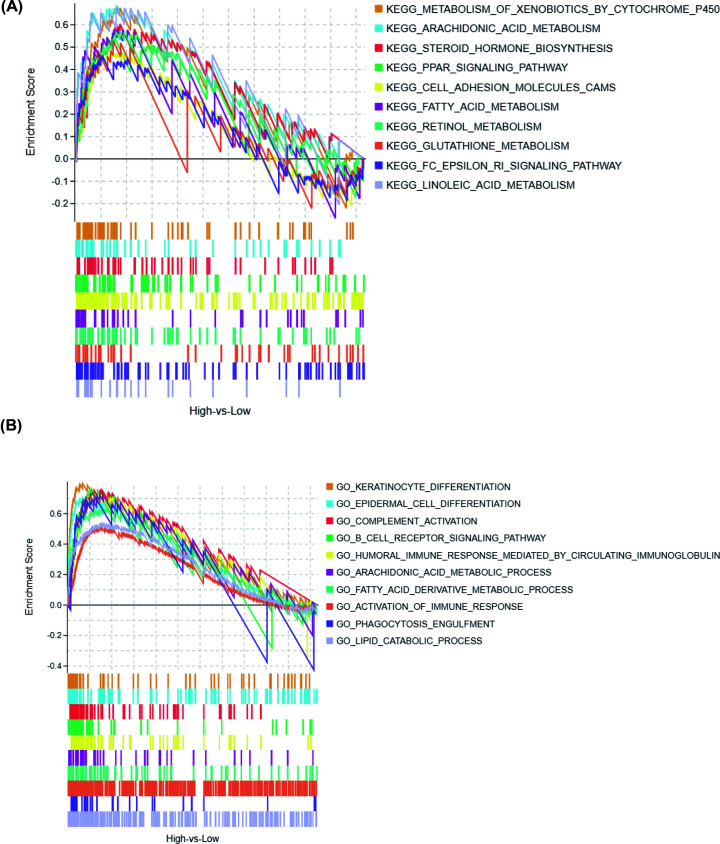
GSEA between high- and low- *FOXD1* expression phenotypes (**A**) KEGG pathways. (**B**) BPs.

FOXD1‐related signaling pathways were analyzed between high- and low-*FOXD1* expression phenotypes through GSEAs. We list the top ten up-regulated signal pathways in the high-expression group of *FOXD1*, including metabolism of xenobiotics by cytochrome P450, arachidonic acid metabolism, steroid hormone biosynthesis, PPAR signaling pathway, cell adhesion molecule (Figure 7A). Moreover, the biological functions were enriched in keratinocyte differentiation, epidermal cell differentiation, complement activation, B-cell receptor signaling pathway, humoral immune response mediated by circulating immunoglobulin in high *FOXD1* phenotype (Figure 7B).

### FOXD1 expression in cell lines and clinical samples

**Figure 8:**
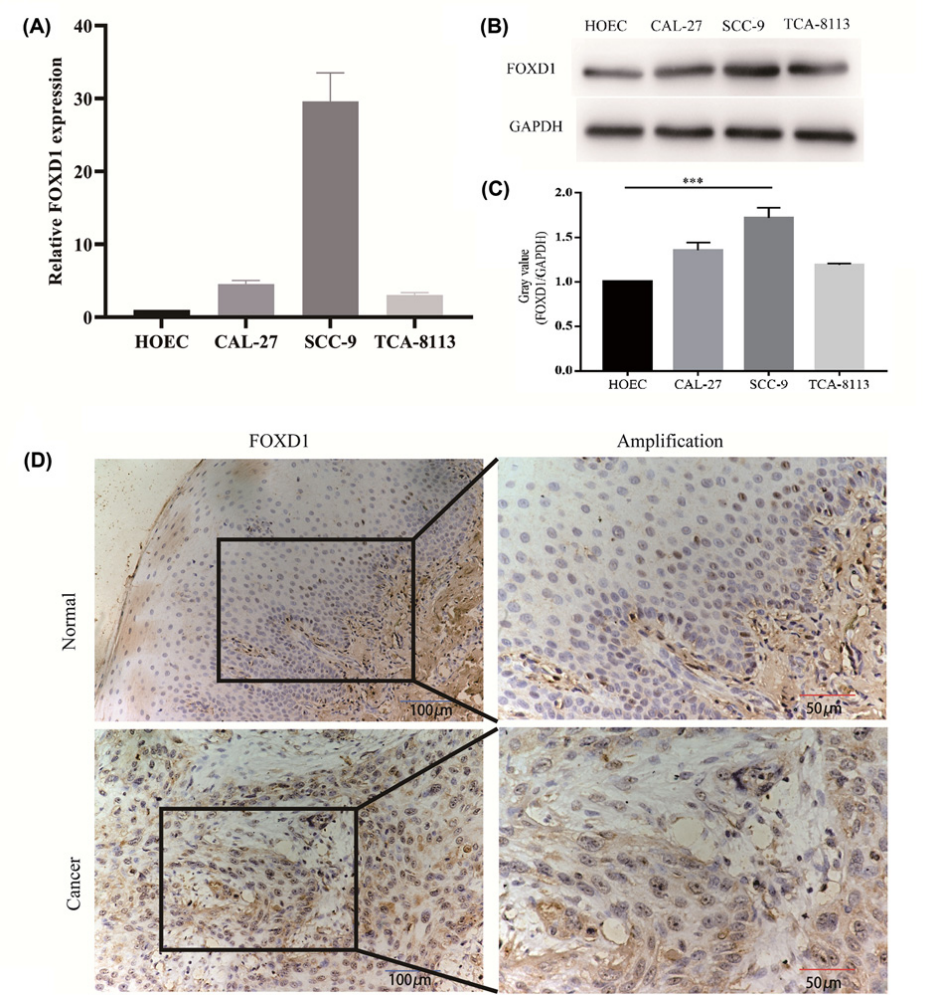
GSEA between high- and low-*FOXD1* expression phenotypes (**A**) Relative FOXD1 mRNA levels in HOEC, CAL-27, SCC-9 and TCA-8113 cell lines. (**B**) Western blot of FOXD1 protein expression in HOEC, CAL-27, SCC-9 and TCA-8113 cell lines. (**C**) Histogram of FOXD1 expression levels in HOEC, CAL-27, SCC-9 and TCA-8113 cell lines. (**D**) FOXD1 expression in HNSC tissue samples and corresponding non‐cancer tissue samples. *** *P*-value<0.001.

To further validate the mRNA expression of *FOXD1*, we assessed the expression levels of *FOXD1* in HNSC cell lines by qRT-PCR. The mRNA levels of FOXD1 were significantly higher in SCC-9 and CAL-27 cell lines compared with those of HOEC cell line (Figure 8A). FOXD1 protein levels were also significantly up-regulated in SCC-9 cell lines (Figure 8B,C). Moreover, FOXD1 expression in HNSC occurred mainly in the nucleus, and FOXD1 expression was significantly increased in cancer tissues compared with corresponding adjacent normal tissues (Figure 8D).

## Discussion

FOX family is an important and complex gene family, comprising diverse cell- and tissue-specific ‘winged-helix’ transcriptional factors [16]. Previous studies demonstrate that FOX family members participate in DNA damage repair, cell proliferation, differentiation, metabolism, apoptosis, tissue homeostasis, and development of immune system [17]. As a consequence, the alterations of FOX expression can influence the tumorigenesis as well as cancer progression. To date, several key FOX gene subfamilies, such as FOXA, FOXC, FOXM, FOXO and FOXP, have been found to be strongly linked to cancer initiation, invasion, metastasis, and drug resistance [18–21]. In the present study, we found that FOXD1 was up-regulated in HNSC tissues at both mRNA and protein levels, and significantly associated with primary tumor site and HPV infection. Moreover, *FOXD1* expression was significantly correlated with OS in HNSC patients. In order to explore the function and molecular mechanism of FOXD1, we analyzed the correlation of *FOXD1* expression and TME, and found that naïve B cells, plasma cells, resting DCs, and activated mast cells were significantly different between low- and high-*FOXD1* expression groups. Moreover, GSEA revealed that *FOXD1* was mainly involved in cancer-related signaling pathway and metabolism-related pathways.

The biological function of FOXD1 in human cancers has not been completely explored. FOXD1, as a newly discovered FOX family transcriptional factor, played a controversial role in varied cancer types. Some studies suggested that FOXD1 was involved in carcinogenesis and functioned as a tumor promoter in many cancer types [8]. Overexpression of FOXD1 could enhance the ability of cell proliferation and chemoresistance in MCF-7 breast cells, whereas silencing of FOXD1 expression attenuate cell proliferation and chemoresistance in MDA-231 cells [10]. Zhang et al. reported that *FOXD1*, as a novel oncogene, promoted the proliferation, migration, invasion, and radio-resistance in nasopharyngeal carcinoma cells [11]. In colorectal cancer patients, FOXD1 expression was up-regulalted and correlated with more invasive phenotype, such as lymphatic metastasis and TNM stage [8]. Tao et al. demonstrated that inhibitior of FOXD1 suppressed cell proliferation, migration and invasion in osteosarcoma cells, while overexpression of FOXD1 promoted osteosarcoma cell proliferation and migration [22]. In prostate cancer, down-regulation of FOXD1 affected the expression of cell cycle control genes and suppressed the androgen-independent growth of 22RV1 cells [13]. However, another study in ovarian cancer indicated that up-regulated FOXD1 could inhibit cell proliferation by inducing cell cycle arrest at G_1_ phase in ovarian cancer cells, and high FOXD1 expression was significantly correlated with favorable prognosis [9]. The results obtained from breast cancer, lung cancer, nasopharyngeal carcinoma, colorectal cancer and prostate cancer are evident, but, the function of FOXD1 in ovarian cancer remain controversial. We performed a pan-cancer anlysis for *FOXD1* at mRNA levels, and found that *FOXD1* was up-regulated in BRCA, CHOL, COAD, ESCA, HNSC, LIHC, LUSC, PRAD and STAD, and down-regulated in KIRC, KIRP, THCA and UCEC. Due to non-available data of normal tissues, there was no comparison in ovarian carcinoma (OV). In view of the up-regulation of *FOXD1* at mRNA levels in TCGA HNSC samples, we validated the *FOXD1* expression in TCGA and GEO datasets. Thus, we speculated that FOXD1 was a tumor promoter in HNSC. Simialr with our result, a recent study reported that FOXD1 up-regulation was related to metastasis and poor clinical outcomes in oral squamous carcinoma [23]. Lin et al. also found that *FOXD1* knockdown dramatically suppressed the colony-forming ability and confered radioresistance by down-regulating the JAK-STAT pathway in oral cancer cells [24]. In the present study, we found that mRNA and protein levels of FOXD1 was up-regulated in HNSC cell lines, and FOXD1 expression was increased in cancer tissues compared with corresponding adjacent normal tissues.

In the last decade, it is clear that FOX family members have potential roles in various aspects of immune regulation, immune homeostasis, and development and differentiation of immune cells [25,26]. FOXO subfamily members participated in the DC activity, CD8^+^ T-cell response, and macrophage activation [27]. FOXJ1 restrains B-cell activation and the maturation of humoral responses to regulate B lymphocyte homeostasis [28]. FOXP1 repressed immune signaling in the central nervous system and contributed to the immune dysfunction in central nervous system development [29]. FOXP3, a molecular marker of Tregs, is highly expressed in lymphoid tissue, and plays a key role in the development and function of Treg cells, representing an important therapeutic target for cancer [30]. FOXN1 is indispensable for thymic epithelial cell differentiation, growth, function, and thymic epithelium cell homeostasis [31,32]. However, the immune function of FOXD1 is little known and its role in TME has not been fully elucidated yet. Lin et al. reported that *Foxd1* coordinated the regulation of the activity of NF-AT and NF-κB, and FOXD1 deficiency could result in multiorgan and systemic inflammation, and exaggerated Th cell-derived cytokine production and T-cell proliferation [33]. HNSC is an immunosuppressive disease, which is characteristic of decreased lymphocyte counts [34], impaired natural killer (NK) cell activity [35], down-regulated antigen-presenting function [36], and abnormality of tumor-infiltrating T lymphocytes [37]. Our results suggested that immune score decreased in HNSC tissues, although the difference was not significant (*P*=0.067). Moreover, we adopt CIBERSORT algorithms to analyze the proportions of immune cell subtypes and found that the naïve B cells, plasma cells, and resting DCs were significantly decreased with *FOXD1* expression increase. Wouters et al. searched Pubmed databse, covering 69 studies and 19 cancer types, and provided the evidence to support a positive role for B cells and plasma cells in antitumor immunity [38]. Moreover, the preoperative DC counts and DC surface molecular expression were impaired in HNSC patients in comparison with healthy controls [39]. Furthermore, we investigated the correlation of FOXD1 expression and changed immune cells, and we found that the proportions of naïve B cells, plasma cells, and resting DCs were negatively correlated with FOXD1 expression; otherwise, the proportion of activated mast cells was positively correlated with FOXD1 expression.

However, our study has some limitation. First, the study was mainly based on TCGA and GEO datasets to investigate the prognostic value of FOXD1, while histological validation was conducted only in a few patients without completed follow-up information. Second, *in vivo* function experiments were lacking. Third, we analyzed the FOXD1-related pathways through GSEA, and there was a lack of relevant mechainsm research.

## Conclusion

Taken together, we identified that up-regulation of *FOXD1* was a potential prognostic marker in HNSC, and was associated with HPV status and HNSC sites. Moreover, *FOXD1* expression played an important role in TME and immune cell infiltration. Hence, further studies are required to validate the role of FOXD1 in HNSC and to improve the understanding of the underlying mechanisms.

## Supplementary Material

Supplementary Figures S1-S2

Tables S1-S3

## Data Availability

The datasets generated and analyzed during the current study are available from the TCGA and GEO database, and the data presented in this manuscript are available from the corresponding author on reasonable request.

## Abbreviations

BRCA: breast invasive carcinoma
CHOL: cholangiocarcinoma
CIBERSORT: Cell-type Identification By Estimating Relative Subsets Of known RNA Transcripts
COAD: colon adenocarcinoma
C-index: concordance index
DC: dendritic cell
ESCA: esophageal carcinoma
ESTIMATE: Estimation of STromal and Immune cells in MAlignant Tumor tissues using Expression data
FOXD1: Forkhead box D1
GEO: Gene Expression Omnibus
GO: gene ontology
GSEA: Gene Set Enrichment Analysis
HNC: head and neck cancer
HNSC: head and neck squamous cancer
HPV: human papillomavirus
KEGG: Kyoto Encyclopedia of Genes and Genomes
KIRC: kidney renal clear cell carcinoma
KIRP: kidney renal papillary cell carcinoma
LIHC: liver hepatocellular carcinoma
LUSC: lung squamous cell carcinoma
NF-AT: nuclear factor of activated T cells
OS: overall survival
PPAR: peroxisome proliferator-activated receptor
STAD: stomach adenocarcinoma
TCGA: The Cancer Genome Atlas
THCA: thyroid carcinoma
TME: tumor microenvironment
TNM: tumor, nodes, metastasis
Treg: regulatory T cells
UCEC: uterine corpus endometrial carcinoma

## References

[1] Torre L.A., Bray F., Siegel R.L., Ferlay J., Lortet-Tieulent J. and Jemal A. (2015) Global cancer statistics, 2012. CA Cancer J. Clin. 65, 87–108 10.3322/caac.21262 25651787

[2] Siegel R.L., Miller K.D. and Jemal A. (2018) Cancer statistics, 2018. CA Cancer J. Clin. 68, 7–30 10.3322/caac.21442 29313949

[3] Levinson R.S., Batourina E., Choi C., Vorontchikhina M., Kitajewski J. and Mendelsohn C.L. (2005) Foxd1-dependent signals control cellularity in the renal capsule, a structure required for normal renal development. Development 132, 529–539 10.1242/dev.01604 15634693

[4] Koga M., Matsuda M., Kawamura T., Sogo T., Shigeno A., Nishida E. et al. (2014) Foxd1 is a mediator and indicator of the cell reprogramming process. Nat. Commun. 5, 3197 10.1038/ncomms4197 24496101

[5] Lin E.E., Sequeira-Lopez M.L. and Gomez R.A. (2014) RBP-J in FOXD1+ renal stromal progenitors is crucial for the proper development and assembly of the kidney vasculature and glomerular mesangial cells. Am. J. Physiol. Renal Physiol. 306, F249–F258 10.1152/ajprenal.00313.2013 24226518 PMC3920017

[6] Herrera E., Marcus R., Li S., Williams S.E., Erskine L., Lai E. et al. (2004) Foxd1 is required for proper formation of the optic chiasm. Development 131, 5727–5739 10.1242/dev.01431 15509772

[7] Li D., Fan S., Yu F., Zhu X., Song Y., Ye M. et al. (2018) FOXD1 promotes cell growth and metastasis by activation of vimentin in NSCLC. Cell. Physiol. Biochem. 51, 2716–2731 10.1159/000495962 30562753

[8] Pan F., Li M. and Chen W. (2018) FOXD1 predicts prognosis of colorectal cancer patients and promotes colorectal cancer progression via the ERK 1/2 pathway. Am. J. Transl. Res. 10, 1522–1530 29887965 PMC5992558

[9] Wang Y., Qiu C., Lu N., Liu Z., Jin C., Sun C. et al. (2018) FOXD1 is targeted by miR-30a-5p and miR-200a-5p and suppresses the proliferation of human ovarian carcinoma cells by promoting p21 expression in a p53-independent manner. Int. J. Oncol. 52, 2130–2142 10.3892/ijo.2018.4359 29620165

[10] Zhao Y.F., Zhao J.Y., Yue H., Hu K.S., Shen H., Guo Z.G. et al. (2015) FOXD1 promotes breast cancer proliferation and chemotherapeutic drug resistance by targeting p27. Biochem. Biophys. Res. Commun. 456, 232–237 10.1016/j.bbrc.2014.11.064 25462566

[11] Zhang Y. and Zhang W. (2020) FOXD1, negatively regulated by miR-186, promotes the proliferation, metastasis and radioresistance of nasopharyngeal carcinoma cells. Cancer Biomark 28, 511–521 10.3233/CBM-191311 32568181 PMC12662369

[12] Wu Q., Ma J., Wei J., Meng W., Wang Y. and Shi M. (2020) FOXD1-AS1 regulates FOXD1 translation and promotes gastric cancer progression and chemoresistance by activating the PI3K/AKT/mTOR pathway. Mol. Oncol. 15, 299–316 10.1002/1878-0261.12728 32460412 PMC7782086

[13] Li X., Jiao M., Hu J., Qi M., Zhang J., Zhao M. et al. (2020) miR-30a inhibits androgen-independent growth of prostate cancer via targeting MYBL2, FOXD1, and SOX4. Prostate 80, 674–686 10.1002/pros.23979 32294305

[14] Gao Y.F., Zhu T., Mao X.Y., Mao C.X., Li L., Yin J.Y. et al. (2017) Silencing of Forkhead box D1 inhibits proliferation and migration in glioma cells. Oncol. Rep. 37, 1196–1202 10.3892/or.2017.5344 28075458

[15] Hartmann S., Bhola N.E. and Grandis J.R. (2016) HGF/Met signaling in head and neck cancer: impact on the tumor microenvironment. Clin. Cancer Res. 22, 4005–4013 10.1158/1078-0432.CCR-16-0951 27370607 PMC6820346

[16] Lam E.W., Brosens J.J., Gomes A.R. and Koo C.Y. (2013) Forkhead box proteins: tuning forks for transcriptional harmony. Nat. Rev. Cancer 13, 482–495 10.1038/nrc3539 23792361

[17] Katoh M., Igarashi M., Fukuda H., Nakagama H. and Katoh M. (2013) Cancer genetics and genomics of human FOX family genes. Cancer Lett. 328, 198–206 10.1016/j.canlet.2012.09.017 23022474

[18] Koo C.Y., Muir K.W. and Lam E.W. (2012) FOXM1: From cancer initiation to progression and treatment. Biochim. Biophys. Acta 1819, 28–37 10.1016/j.bbagrm.2011.09.004 21978825

[19] Golson M.L. and Kaestner K.H. (2016) Fox transcription factors: from development to disease. Development 143, 4558–4570 10.1242/dev.112672 27965437 PMC5201025

[20] Bach D.H., Long N.P., Luu T.T., Anh N.H., Kwon S.W. and Lee S.K. (2018) The dominant role of forkhead box proteins in cancer. Int. J. Mol. Sci. 19, 3279 10.3390/ijms19103279 PMC6213973 30360388

[21] Laissue P. (2019) The forkhead-box family of transcription factors: key molecular players in colorectal cancer pathogenesis. Mol. Cancer 18, 5 10.1186/s12943-019-0938-x 30621735 PMC6325735

[22] Tao J., Cong H., Wang H., Zhang D., Liu C., Chu H. et al. (2018) MiR-30a-5p inhibits osteosarcoma cell proliferation and migration by targeting FOXD1. Biochem. Biophys. Res. Commun. 503, 1092–1097 10.1016/j.bbrc.2018.06.121 29936179

[23] Li Z., Yan T., Wu X., Zhang W., Li J., Wang L. et al. (2020) Increased expression of FOXD1 is associated with cervical nodes metastasis and unfavorable prognosis in oral squamous cell carcinoma. J. Oral Pathol. Med. 49, 1030–1036 10.1111/jop.13098 32808339

[24] Lin C.H., Lee H.H., Chang W.M., Lee F.P., Chen L.C., Lu L.S. et al. (2020) FOXD1 repression potentiates radiation effectiveness by downregulating G3BP2 expression and promoting the activation of TXNIP-related pathways in oral cancer. Cancers (Basel) 12, 2690 10.3390/cancers12092690 PMC7563336 32967107

[25] Zaiss D.M.W. and Coffer P.J. (2018) Forkhead box transcription factors as context-dependent regulators of lymphocyte homeostasis. Nat. Rev. Immunol. 18, 703–715 10.1038/s41577-018-0048-9 30177790

[26] Skinner J., Greene R.A. and Stuart B. (1997) Puerperal ovarian vein thrombosis in a triplet pregnancy complicated by a single intrauterine death. J. Obstet. Gynecol. 17, 585 10.1080/01443619768704 15511968

[27] Cabrera-Ortega A.A., Feinberg D., Liang Y., Rossa C. Jr and Graves D.T. (2017) The role of forkhead box 1 (FOXO1) in the immune system: dendritic cells, T cells, B cells, and hematopoietic stem cells. Crit. Rev. Immunol. 37, 1–13 10.1615/CritRevImmunol.2017019636 29431075 PMC6085137

[28] Lin L., Brody S.L. and Peng S.L. (2005) Restraint of B cell activation by Foxj1-mediated antagonism of NF-kappa B and IL-6. J. Immunol. 175, 951–958 10.4049/jimmunol.175.2.951 16002694

[29] Tang B., Becanovic K., Desplats P.A., Spencer B., Hill A.M., Connolly C. et al. (2012) Forkhead box protein p1 is a transcriptional repressor of immune signaling in the CNS: implications for transcriptional dysregulation in Huntington disease. Hum. Mol. Genet. 21, 3097–3111 10.1093/hmg/dds132 22492998 PMC3384380

[30] Nik Tavakoli N., Hambly B.D., Sullivan D.R. and Bao S. (2008) Forkhead box protein 3: essential immune regulatory role. Int. J. Biochem. Cell Biol. 40, 2369–2373 10.1016/j.biocel.2007.10.004 18037337

[31] Zuklys S., Handel A., Zhanybekova S., Govani F., Keller M., Maio S. et al. (2016) Foxn1 regulates key target genes essential for T cell development in postnatal thymic epithelial cells. Nat. Immunol. 17, 1206–1215 10.1038/ni.3537 27548434 PMC5033077

[32] Zhang Z., Burnley P., Coder B. and Su D.M. (2012) Insights on FoxN1 biological significance and usages of the “nude” mouse in studies of T-lymphopoiesis. Int. J. Biol. Sci. 8, 1156–1167 10.7150/ijbs.5033 23091413 PMC3477685

[33] Lin L. and Peng S.L. (2006) Coordination of NF-kappaB and NFAT antagonism by the forkhead transcription factor Foxd1. J. Immunol. 176, 4793–4803 10.4049/jimmunol.176.8.4793 16585573

[34] Kuss I., Hathaway B., Ferris R.L., Gooding W. and Whiteside T.L. (2004) Decreased absolute counts of T lymphocyte subsets and their relation to disease in squamous cell carcinoma of the head and neck. Clin. Cancer Res. 10, 3755–3762 10.1158/1078-0432.CCR-04-0054 15173082

[35] Dasgupta S., Bhattacharya-Chatterjee M., O’Malley B.W. Jr and Chatterjee S.K. (2005) Inhibition of NK cell activity through TGF-beta 1 by down-regulation of NKG2D in a murine model of head and neck cancer. J. Immunol. 175, 5541–5550 10.4049/jimmunol.175.8.5541 16210663

[36] Ferris R.L., Whiteside T.L. and Ferrone S. (2006) Immune escape associated with functional defects in antigen-processing machinery in head and neck cancer. Clin. Cancer Res. 12, 3890–3895 10.1158/1078-0432.CCR-05-2750 16818683

[37] Whiteside T.L. (1998) Immune cells in the tumor microenvironment. Mechanisms responsible for functional and signaling defects. Adv. Exp. Med. Biol. 451, 167–171 10.1007/978-1-4615-5357-1_27 10026868

[38] Wouters M.C.A. and Nelson B.H. (2018) Prognostic significance of tumor-infiltrating B cells and plasma cells in human cancer. Clin. Cancer Res. 24, 6125–6135 10.1158/1078-0432.CCR-18-1481 30049748

[39] Ma X.J., Pan X.L., Lv Z.H., Xu F.L., Liu D.Y., Lei D.P. et al. (2009) Therapeutic influence on circulating and monocyte-derived dendritic cells in laryngeal squamous cell carcinoma patients. Acta Otolaryngol. 129, 84–91 10.1080/00016480802020459 18607895

