# Supplementary material for: FOXD1 expression in head and neck squamous carcinoma: a study based on TCGA, GEO and meta-analysis
Source: Biosci Rep. 2021 Jul 28;41(7):BSR20210158. doi: 10.1042/BSR20210158 (PMC8319493; doi:10.1042/BSR20210158)
Supplement: Supplementary Figures S1-S2 [file BSR-2021-0158_supp.pdf]

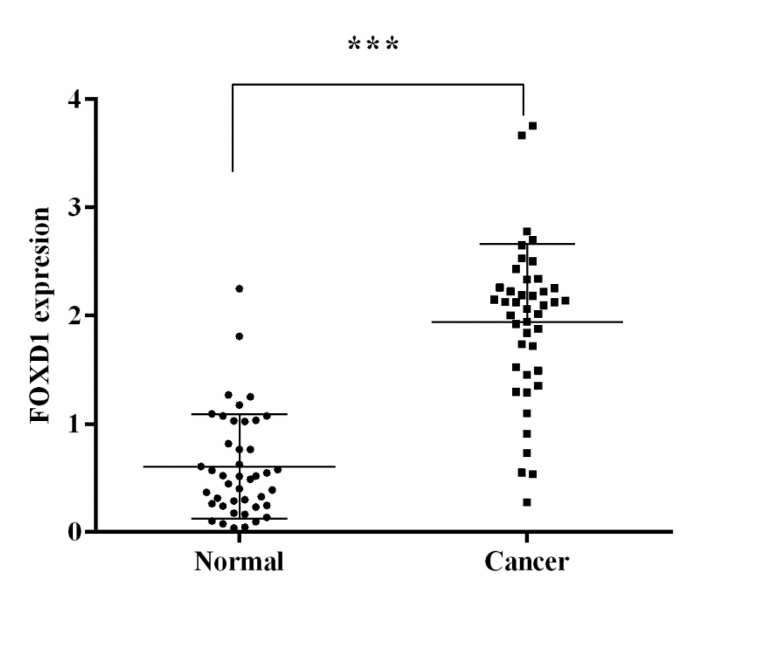

Figure S1. FOXD1 expression between paired HNSC and normal tissues (N=43 pairs).

(A)

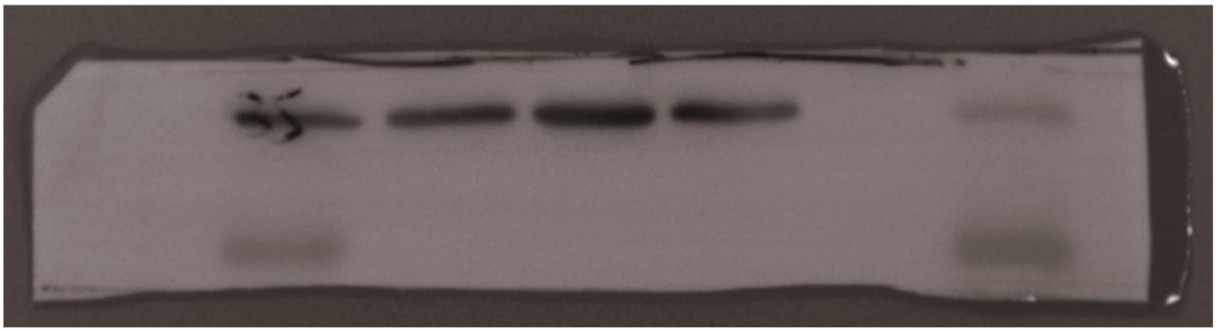

(B)

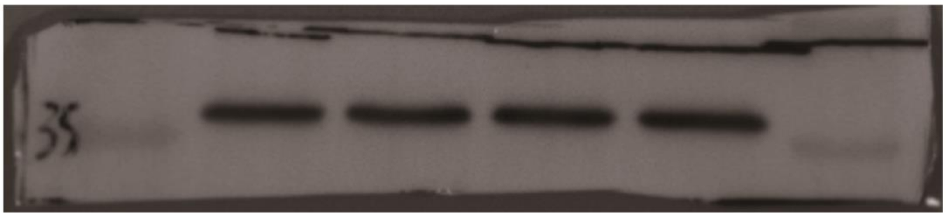

Figure S2. Original picture of western blot (from left to right: HOEC, CAL-27, SCC-9 and TCA-8113 cell lines). (A) FOXD1 expression; (B) GAPDH expression
