# Supplementary material for: FOXD1 expression in head and neck squamous carcinoma: a study based on TCGA, GEO and meta-analysis
Source: Biosci Rep. 2021 Jul 28;41(7):BSR20210158. doi: 10.1042/BSR20210158 (PMC8319493; doi:10.1042/BSR20210158)
Supplement: Tables S1-S3 [file BSR-2021-0158_supp1.zip › BSR-2021-0158_suppST3.docx]

Table S3. Clinical characteristics of HNSC patients

| Parameters | Number | Percentage |
| --- | --- | --- |
| Age |  |  |
| ≤60 years | 211 | 47.0% |
| >60 years | 238 | 53.0% |
| Gender |  |  |
| Male | 327 | 72.8% |
| Female | 122 | 27.2% |
| Distant metastasis |  |  |
| Without metastasis | 444 | 98.9% |
| With metastasis | 5 | 1.1% |
| Clinical stage |  |  |
| Stage I | 17 | 3.8% |
| Stage II | 86 | 19.2% |
| Stage III | 96 | 21.3% |
| Stage IV | 250 | 55.7% |
| N stage |  |  |
| N0 | 226 | 50.3% |
| N1 | 78 | 17.4% |
| N2 | 138 | 30.7% |
| N3 | 7 | 1.6% |
| T stage |  |  |
| T1 | 30 | 6.7% |
| T2 | 127 | 28.3% |
| T3 | 124 | 27.6% |
| T4 | 168 | 37.4% |
| Grade |  |  |
| G1 | 53 | 11.8% |
| G2 | 279 | 62.1% |
| G3 | 117 | 26.1% |
| Tumor site |  |  |
| Oral | 340 | 75.7% |
| Larynx | 109 | 24.3% |
| HPV infection |  |  |
| Negative | 61 | 13.6% |
| Positive | 13 | 2.9% |
| NA | 375 | 83.5% |
